# Interest of body composition assessment in the accuracy of the diagnosis of undernutrition in the older population in Senegal: A cross-sectional and prospective study

**DOI:** 10.1016/j.jarlif.2025.100030

**Published:** 2025-10-23

**Authors:** Sandra J Bitchoka Mbea, Maïmouna Touré, Claude Stephan Ohandza, Mamadou Coume

**Affiliations:** a Geriatrics Department, Fann/Dakar University Hospital, PO box: 45690, Senegal; b Laboratory of Physiology and Functional Explorations, Faculty of Medicine, Pharmacy and Odontostomatology, Cheikh Anta Diop University, Fann, PO box: 5005, Dakar, Senegal; c Department of Infectious diseases and Tropical Medicine, Fann/Dakar University Hospital, PO box: 45690, Senegal; d Department of Geriatrics, Faculty of Medicine, Pharmacy and Odontostomatology, Cheikh Anta Diop University, Fann, PO box: 5005, Dakar, Senegal

**Keywords:** Body composition, Undernutrition, Elderly people

## Abstract

**Introduction:**

In the absence of consensus on the diagnostic criteria for undernutrition in the elderly, there is a significant number of missing cases of disease in this target, particularly in areas with limited resources, including Senegal. We wanted to contribute to a better diagnostic approach to undernutrition in the elderly, by determining the relevance of body composition assessment using BIA.

**Design:**

Cross-sectional and prospective study.

**Setting and Participants:**

Individuals aged ≥ 60 years old, attending outpatient consultations at Fann University Hospital and the Retirement Provident health Center (IPRES), who were able to stand for 3 min without technical and/or human assistance were included.

**Methods:**

We used the Mini Nutritional Assessment (MNA) and Body Mass Index (BMI) to determine the prevalence of undernutrition and a Tanita BC 601® brand body composition monitor to assess body composition. Data were analyzed using SPSS version 29.0.

**Results:**

We included 73 individuals with a mean age of 72.48 ± 7.23 years old, predominantly male, with a sex ratio of 1.43. Undernutrition, the main geriatric syndrome, affected 46.6 % of the study subjects according to the MNA and/or BMI, including two- thirds in men. The mean lean mass index was 16.07 ± 3.03 kg/m² in men and 15.53 ± 2.51 kg/m² in women. It was significantly lower in cases of malnutrition, with an average of 14.38 ± 2.82 kg/m² among malnourished elderly individuals.

**Conclusion:**

Malnutrition in the elderly is best assessed early by a combination of diagnostic tools including MNA, BMI and lean mass index assessed by BIA.

## 1 Introduction

Undernutrition is defined as a pathological condition that results from an imbalance between insufficient intake and increased physiological needs of the body [1]. In the absence of a consensus on the diagnostic criteria for undernutrition in the elderly, there is a great variability in the prevalence of this geriatric syndrome on the African continent. In addition to the context, this can also be explained by the diversity of diagnostic methods [2]. For example, in 2006 in Dakar, researchers found prevalences of 8.73 % and 0.97 % based on the MNA and BMI, respectively, within the same study population [3]. As a result, data on undernutrition in the elderly may include missing cases [4]. In addition, recent revisions of the diagnostic criteria for undernutrition by international organizations such as the Global Leadership Initiative on Malnutrition (GLIM) and the Haute Autorité de Santé (HAS) have excluded MNA from these criteria, particularly in the elderly population [5,6]. Currently, the elements that define the phenotypic criteria for undernutrition in the elderly include weight loss, body mass index and sarcopenia [6]. The latter is defined as a decrease in muscle strength and muscle mass, ideally measured by Dual-energy X-ray absorptiometry (DEXA) [6]. This confirms the presence of altered body composition, characterized by a decrease in lean mass and/or an increase in fat mass in cases of undernutrition among the elderly. Thus, body composition assessment is an essential tool for nutrition specialists and geriatricians. Among the available methods; BMI, waist circumference, and arm circumference are widely used due to their simplicity, low cost, and the fact that they do not involve radiation exposure [7]. Bio-impedance measurement (BIA) is a newer, fast, relatively inexpensive, non-invasive, and reproducible method. It is portable and well-suited for use in older African populations, making it a reliable alternative to DEXA [[8], [9], [10]]. However, to the best our knowledge, no previous study has determined reference values for body composition parameters for the elderly population in West Africa [15]. Data from Asian and Western studies are not transferable to our context. In Senegal, a pioneer in the field of geriatrics in sub-Saharan Africa, no study has, to our knowledge, assessed body composition as part of the nutritional evaluation of elderly individuals. With the aim of contributing to a better diagnostic approach to undernutrition in older adults in our setting, we undertook this study, whose overall objective was to determine the relevance of body composition assessment in improving the accuracy of undernutrition diagnosis in the elderly.

## 2 Methods

### 2.1 Study design and population

A cross-sectional and prospective study was conducted over a 12-month period (June 2023 to May 2024) at the Fann University Hospital Center (CHNU) and the Retirement Provident health Center in Dakar, Senegal (IPRES). These two institutions serve as major outpatient care and research hubs for the elderly population, with consultations led by geriatricians, gerontologists, and residents in training. Hospital admissions are limited to the CHNU-Fann site, where a clinical nutritionist is also available. Within routine clinical practice, comprehensive geriatric assessments are systematically performed for nearly all outpatients and all inpatients, using internationally validated tools. Nutritional status is assessed using the MNA, in accordance with the 2007 guidelines of the French Haute Autorité de Santé.

The study included 73 individuals aged ≥ 60 years old, consecutively recruited during outpatient consultations. Participants were required to be able to stand unassisted for at least three minutes, without technical or human support. Exclusion criteria were as follows:

- incomplete survey forms, typically in patients with major neurocognitive disorders or hyperactive delirium, resulting in non-cooperation;
- any progressive disease associated with sustained hypercatabolic states;
- clinical signs of fluid overload (e.g., edema);
- presence of a cardiac pacemaker;
- morbid obesity (BMI > 40 kg/m²).

### 2.2 Data collection

Data were collected using a pre-tested semi-structured interview guide and validated tools for comprehensive geriatric assessment. During participant recruitment, all data collection instruments were systematically translated into Wolof to facilitate understanding.

2.2.1. Sociodemographic characteristics: age, sex, marital status, educational level, and living environment.
2.2.2. Comorbidities and medical/surgical history : hypertension, diabetes, psychological stress, recent illnesses (within the past three months), depression, early-stage neurodegenerative disorders, oral health issues (including use of dental prostheses), gastrointestinal disorders, and the number of medications taken daily, among others.
2.2.3. Dietary habits (as part of the global assessment in the full MNA): number of meals per day, most frequently consumed foods, daily intake of plant- and animal-based proteins, fruit consumption, daily water intake, and perceived satisfaction with one's nutritional status.
2.2.4. Data from the multidimensional geriatric assessment, including the evaluation of :
  ✓ Frailty, using the Fried’s criteria;
  ✓ Autonomy and functional independence, assessed by the Activities of Daily Living (ADL) and Instrumental Activities of Daily Living (IADL) scales;
  ✓ Depression, screened using the Mini Geriatric Depression Scale (Mini-GDS);
  ✓ Cognitive function, evaluated with the Senegal Test;
  ✓ Nutritional status, assessed using the full MNA, scored out of 30 points, classifying older adults as follows:
    - Malnourished for an MNA score < 17 points,
    - At risk of undernutrition for a score between 17 and 23.5 points
    - Normal nutritional status for a score ≥ 24 points.
2.2.5. Anthropometric parameters: weight, height, BMI, mid-upper arm circumference, and calf circumference.

The measurement of anthropometric parameters was carried out according to the following references :

o Height was taken from the individual’s identification document, provided it had been measured within the previous two years. Otherwise, it was measured using a measuring tape, to the nearest centimeter.
o Mid-upper arm circumference was measured to the nearest 0.1 cm at the midpoint between the acromion and the olecranon process on the non-dominant arm, while the participant held the forearm in a horizontal position.
o The largest calf circumference was measured to the nearest 0.1 cm between the ankle and the knee, using a non-elastic measuring tape. Measurements were taken with the participant seated, the knee flexed at 90°, and the tape applied in close contact with the skin without compression.
2.2.6. Body composition parameters: body fat percentage, muscle mass, bone mass, total body water, and visceral fat level.

Fat-free mass (FFM) and the Fat-Free Mass Index (FFMI) were calculated using the following formulas :

**Fat-Free Mass (FFM) = Weight × [1 –(Body Fat Percentage / 100)]**

**Fat-Free Mass Index (FFMI) = FFM / Height²**

Body composition was assessed using a Tanita BC 601® digital bioelectrical impedance scale.

Measurements were performed in accordance with the manufacturer's instructions, at least three hours after the last meal and/or fluid intake. Participants were weighed wearing only light clothing, excluding jackets, shoes, belts, and jewellery. Prior to each measurement, the device was calibrated by inputting the participant’s age, sex, and height.

Participants were then instructed to stand barefoot on the metal footplates and to grasp a pair of electrodes attached to a handheld device, with their arms extended horizontally at chest level. Potential confounding conditions that could affect the accuracy of the measurements were also controlled.

In this study, undernutrition was defined as a state of imbalance between nutritional needs and intake, characterized by a total MNA score ≤ 17 and/or a Short Form MNA (MNA-SF) score < 7, and/or a BMI < 22 kg/m². Based on the Fat-Free Mass Index (FFMI), undernutrition was also identified using threshold values of < 17 kg/m² for men and < 15 kg/m² for women.

### 2.3 Statistical analysis

Data were analyzed using SPSS software, version 29.0. Continuous variables were expressed as means ± standard deviations, and categorical variables as frequencies and percentages. Student’s *t*-test and ANOVA were used for group comparisons. The Spearman correlation test, as well as the Chi-square test or Fisher’s exact test, were used for bivariate analysis between categorical variables. A significance level of α < 0.05 was considered statistically significant.

## 3 Results

### 3.1 Socio-demographic characteristics

The study population included 73 participants, with a mean age of 72.48 ± 7.23 years. A male predominance was observed (58.9 %, sex ratio: 1.43).

### 3.2 Clinical features and comorbidities

Hypertension was the most common comorbidity (64.4 %), followed by type 2 diabetes (30.1 %). Among other conditions potentially influencing the nutritional status of participants, 28.8 % of the elderly subjects suffered from rheumatologic disorders and 26 % had digestive pathologies. The rate of dental prosthesis use was 45.2 %, reflecting partial rehabilitation of oral health issues. Nine cases of neurodegenerative diseases were recorded, notably mild cognitive impairment cases without formal diagnosis. Although information on specific classes of medications used by the elderly was not available, most were antihypertensive drugs, including diuretics and Angiotensin Converting Enzyme (ACE) inhibitors.

### 3.3 Geriatric conditions

The overall prevalence of undernutrition was 46.6 %, with severe undernutrition (BMI < 20 kg/m²) observed in 21.9 % of participants. According to the MNA, 17.8 % of the elderly were classified as malnourished (score < 17), while 34.2 % were at risk of undernutrition (score between 17 and 23.5). There was 39.7 % of undernutrition according to BMI (Table I).

**Table I:** Distribution by geriatric conditions.

Geriatric Assessment Test Scores	Count (N)	Percentages ( %)
Evaluation of frailty by the FRIED score		
< 3	41	56.2
≥ 3	32	43.8
KATZ ADL		
6	53	72.6
5 - 5.5	7	9.6
4 - 4.5	6	8.2
3 –3.5	3	4.1
1 –1.5	3	4.1
0 –0,5	1	1.4
LAWTON’s IADL (Female)		
7 –8	17	56.7
5 –6	6	20
3 –4	3	10
0 –2	4	13.3
LAWTON's IADL (male)		
4	33	76.7
3	2	4.7
2	4	9.3
0 –1	4	9.3
Evaluation of cognitive functions by the SENEGAL test		
≥ 30	40	54.8
[22- 30 [	27	37.0
< 22	6	8.2
Searching for suspected cases of depression by the Mini GDS		
Positive	6	8.2
Negative	67	91.8
Scores of MNA short form		
[[12], [13], [14]]	32	43.8
[[8], [9], [10], [11]]	27	37
[7]	14	19.2
Scores of MNA full Version		
≥ 23.5	35	48
]17- 23.5]	25	34.2
≤ 17	13	17.8
Scores of BMI (kg/m²)		
< 22	29	39.7
[22 –30]	32	43.8
> 30	12	16.5

### 3.4 Eating habits

The majority of participants (67.1 %) ate at least three meals per day. Rice was the most frequently consumed food (86.3 %), and 82.2 % of participants had daily access to animal protein. However, only 43.8 % consumed fruit daily. 64.4 % of seniors reported being satisfied with their nutritional status (Table II).

**Table II:** Eating habits.

Eating habits	Workforce (N)	Percentages ( %)
Number of daily meals		
≥ 3	49	67.1
2	23	31.5
1	1	1.4
Most frequently consumed meal		
Rice	63	86.3
Vegetables	10	13.7
Daily consumption of milk, cheese or other dairy products		
Yes	45	61.6
Not	28	38.4
Daily consumption of fish and/or meat		
Yes	60	82.2
Not	13	17.8
Consumption of proteins from other sources (eggs, lentils)		
Yes	33	45.2
Not	40	54.8
Daily Fruit Consumption		
Yes	41	56.2
Not	32	43.8
Daily drink consumption (including water, tea, fruit juice)		
>5 glasses	45	61.6
Between 3 and 5 glasses	14	19.2
<3 glasses	14	19.2
Nutrition satisfaction survey (refers to quality and quantity)		
Yes	47	64.4
Not	15	20.5
Don't know	11	15.1

### 3.5 Body composition

**Fig. 1:**
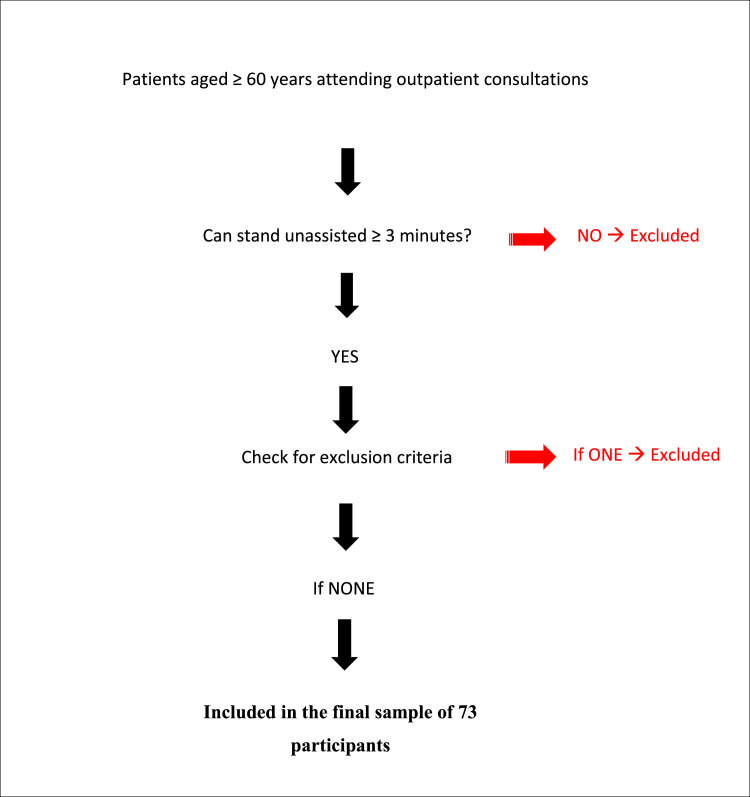
Flowchart showing the inclusion and exclusion criteria of participants.

The mean weight was 68.13 ± 16.34 kg, and the mean BMI was 24.38 ± 6.06 kg/m². The average body fat percentage was 33.43 ± 11.44 %, while the average muscle mass was 42.98 ± 7.31 kg. The mean LMI was 15.85 ± 2.82 kg/m², with significantly lower values observed in malnourished participants (14.38 ± 2.82 kg/m²) (Fig. 1).

#### 3.5.1 Gender variations

Sex significantly affects body fat percentage, muscle mass, and bone mass with p-values respectively at *p* = 0.006; *p* = 0.036 and *p* = 0.006.

**Fig. 2:**
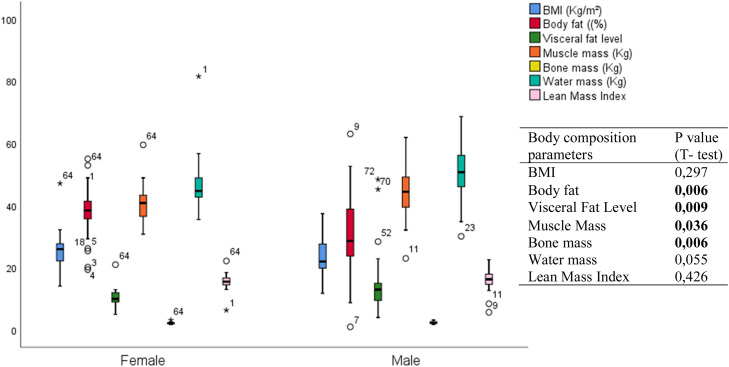
Means of body composition parameters in the study population: distribution by sex.

Males had significantly higher muscle mass (44.46 ± 7.81 kg) and bone mass (2.42 ± 0.35 kg) than females (40.80 ± 5.99 kg and 2.20 ± 0.29 kg, respectively) (Fig. 2).

**Age variations**

**Fig. 3:**
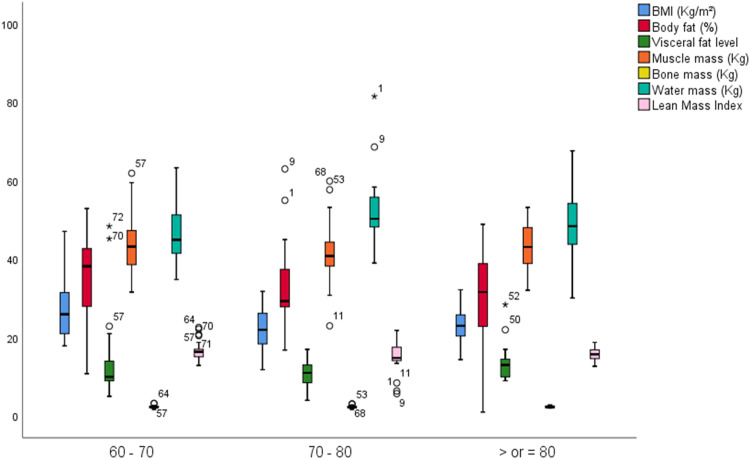
Mean body composition parameters of the study population by age group.

The 70–80-year age group displayed a globally more altered body composition profile compared to the two extreme age groups in our sample, as shown in Fig. 3. We used the ANOVA test to identify differences across the various age groups with a statistical significance *p* < 0.05.

(a) Only BMI showed significant variation with age, with ANOVA p-value of 0.019, respectively.

#### 3.5.2 Changes in body composition by nutritional status

**Fig. 4:**
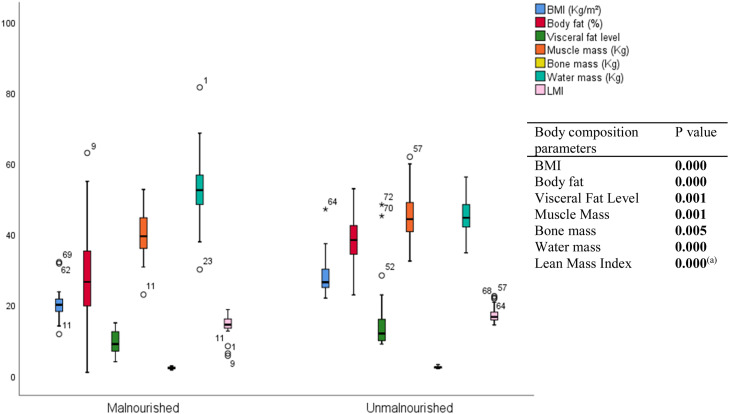
Mean body composition parameters in the study population : distribution by nutritional status.

Overall, there was a highly significant decrease in body composition parameters among malnourished older adults. Fig. 4 shows a progressive increase in the different body compartments with normal nutritional status, with a statistically significant difference between the two groups; except for total body water.

(a) The mean lean mass index in this group was 14.38 ± 2.82 versus 17.04 ± 2.22 in the group of PAs with normal nutritional status. The difference between these two groups was statistically highly significant (p value < 0.0001).

#### 3.5.3 Correlation between body composition parameters

Overall, there was a significant correlation between BMI and lean mass index, with a Spearman correlation coefficient of 0.734 and a p-value of 0.000.

In the absence of undernutrition, BMI was significantly correlated with weight (correlation coefficient = 0.809; *p* = 0.000), body fat percentage (*r* = 0.600; *p* = 0.000), visceral fat level (*r* = 0.453; *p* = 0.004), muscle mass (*r* = 0.326; *p* = 0.043), and bone mass (*r* = 0.329; *p* = 0.041). A significant negative correlation was observed between BMI and total body water (Spearman *r* = −0.681).

Among malnourished older adults, the only persistent correlation was between muscle mass and bone mass.

#### 3.5.4 Prevalence of undernutrition in the study population by lean body mass index

**Fig. 5:**
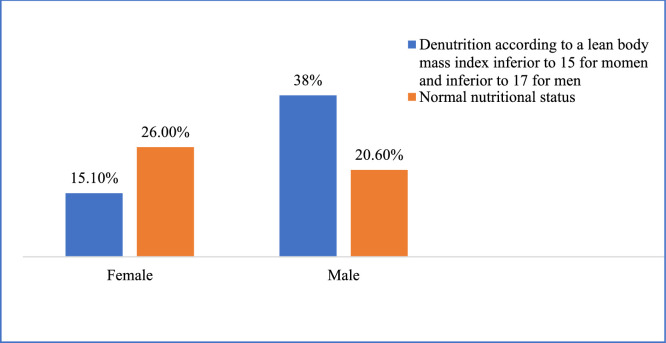
Distribution of the population by sex according to the lean body mass index.

Among men, 28 (38.3 %) had a fat-free mass index (FFMI) below 17 kg/m², and among women, 11 (15.1 %) had an FFMI below 15 kg/m², as shown in Fig. 5.

## 4 Discussion

Undernutrition, a condition closely linked to poverty, remains a major public health concern in Africa and particularly in Senegal, as evidenced by the high prevalence reported by Ba et al. (68.3 %) in a hospital setting in Dakar [11]. Among the priority actions to be undertaken in this population to combat undernutrition, it is essential to reduce the number of undiagnosed or unrecorded cases. This requires reaching a consensus on a diagnostic approach that is both effective and adapted to the local context. In this study, the MNA tended to underestimate the prevalence of undernutrition compared to BMI and the lean mass index. Similar findings were reported by Woldekidan et al. in Ethiopia [12]. Conversely, André et al. in the Democratic Republic of Congo observed a tendency of the MNA to overestimate undernutrition in the elderly population, reporting prevalences of 54 % according to the MNA and 31 % based on BMI [13]. This same trend was also noted by Cascio et al. and Diagne et al. [3,14].. Given the limited availability of resources for the management of this geriatric syndrome, preventive measures offer a more effective alternative. Among these, the assessment and monitoring of body composition may play a critical role. In our context, where access to the reference method for assessing body composition is limited, BIA using an impedance scale may represent a reliable alternative [15]. The decline in muscle mass, reflected by a decrease in the LMI, is considered almost pathognomonic of undernutrition [16]. Or, nous n’avons pas trouvé d’étude précédente sur le sujet. The results of this pioneer study could be used as reference values in Sub-Saharan Africa. In fact, in this sample, 28 men (38.3 %) had an LMI below 17 kg/m² and 11 women (15.1 %) had an LMI below 15 kg/m². Based on these thresholds, the prevalence of undernutrition in the study population would be 53.42 % (39 cases). A marked reduction in LMI was observed in the undernourished group (mean LMI of 14.38 ± 2.82 kg/m²), consistent with findings in the literature. BIA appears to characterize undernutrition in older adults primarily through reduced muscle mass. Therefore, it could contribute to early diagnosis and the assessment of undernutrition severity in older individuals, particularly in settings where the MNA tends to underestimate its prevalence. The lower mean LMI observed in men (16.04 kg/m²) may be explained by the higher prevalence of undernutrition in this subgroup. Variations in lean mass are primarily reflected by changes in muscle mass and, to a lesser extent, by changes in bone mass. The average muscle and bone masses in our study were 42.98 ± 7.31 kg and 2.33 ± 0.34 kg, respectively. Muscle mass was lower than that reported by Galmés-Panadés et al. (56.5 ± 6.56 kg) [17]. This discrepancy may be attributed to the fact that their study population was generally younger (mean age of 65.3 years), and it is well established that muscle mass decreases with age, albeit less markedly in men. Additionally, the difference in methodology; DEXA in their study versus BIA in ours, could explain the variation in muscle mass measurements. Muscle mass varied significantly according to sex (*p* = 0.036), being higher in men, in line with the literature. This finding lends further credibility to the BIA performed with the Tanita BC 601® device used in our study. However, we did not observe the expected age-related decline in muscle mass within our sample. There was a strong and significant association between reduced muscle mass and nutritional status, as previously documented in multiple studies [8]. Since muscle loss is frequently accompanied by a decrease in skeletal muscle mass, our study population appears to be at high risk of sarcopenia, and therefore at increased risk of functional decline and loss of autonomy [18]. This highlights the need to prioritize early screening for malnutrition in older adults. The mean bone mass in our sample was 2.33 ± 0.34 kg, with a significant difference between sexes. Our results show that bone mass was higher in older men compared to women, which is consistent with findings in the literature. We also observed a significant association between bone mass and nutritional status, as previously reported. Malnutrition can lead to vitamin D and calcium deficiencies, thereby increasing the risk of falls and fractures, particularly in women. This association between malnutrition and fall risk was demonstrated by Diagne et al. [3]. Weight and BMI were higher in the female population compared to the male population, as previously reported, although this difference was not statistically significant. A slight overweight status was observed among women, with a mean BMI slightly above 25 kg/m², corresponding to a higher body fat percentage in this group. Nonetheless, this observation does not preclude the risk of malnutrition in women. The average body fat percentage recorded in our study population was 33.43 ± 11.44 %. This value is lower than that reported by Galmés-Panadés et al. in Brazil, who found a higher average of 40.5 ± 6.90 % in a younger population with a higher mean BMI (>32 kg/m²) [17]. The mean BMI was lower in our study population. Although an increase in fat mass is expected as part of normal aging, we observed a decline in fat mass with increasing age in our sample. This decrease in body fat percentage was paralleled by a reduction in BMI. Such findings may be explained by the significant impact of malnutrition on fat mass in our study. Indeed, we found a statistically significant difference in body fat percentage between malnourished and non-malnourished older adults (*p* = 0.0001). However, other studies have reported either an increase or stability in fat mass in cases of malnutrition [9,19]. These discrepancies highlight that changes in fat mass alone are insufficient to determine nutritional status; a comprehensive analysis of all body composition parameters is essential. We also observed a higher total body water content among malnourished older adults compared to their well-nourished counterparts. Nevertheless, none of the study participants showed clinical signs of extracellular dehydration or fluid overload. It is well established that malnourished individuals often have reduced muscle mass and altered body composition, which may influence water distribution and retention.

## 5 Strengths and limitations

### 5.1 Strengths

- Relevance in the local context in the absence of a database on body composition parameters for elderly people in Senegal and West Africa
- Fundamental for the development of future research infrastructure on geriatric nutrition in sub-Saharan Africa. Although cross-sectional in design, this work lays the groundwork for longitudinal studies and offers practical advice on data collection strategies, recruitment challenges, and the integration of body composition measurements into routine geriatric care.
- Complementary to current diagnostic methods for malnutrition in older adults (MNA, BMI) and early detection
- Guides targeted, personalised nutritional management strategies
- Easy reproducibility of the measurement method, non-operator dependent. Potential confounding factors that could affect the accuracy of the measurements were also controlled, in particular situations underlying a state of dehydration (presence of fever, diarrhoea); the absence of pacemakers and the removal of other metal objects (watches, other jewellery, coins); the temperature of the examination room at 24 °C.

### 5.2 Limitations

- Limited sample size and potential selection bias because the two selected hospitals are in the most populated area of Senegal.
- Cost and access to key equipment which leads to dependence on available measurement tools, because in fact, we used our own resources to purchase a scale for this study.
- Lack of comparative data in our context: West Africa and Africa. The Senegalese context, with its social, cultural and economic particularities, may make it difficult to directly apply international diagnostic standards and this also complicate comparisons with other studies conducted in different contexts.

## 6 Conclusion

This study highlights a substantial prevalence of undernutrition among elderly individuals in our setting. This prevalence appears to be underestimated by the MNA, which remains the most used international screening tool in African contexts. Body composition analysis provided a more accurate assessment by compensating for this underestimation, allowing for a better characterization and grading of undernutrition. BIA, used alongside MNA and BMI, enables early detection of undernutrition in older adults, particularly those at risk of sarcopenia and loss of autonomy. In settings where DEXA is not accessible, BIA offers a reliable and practical alternative for the screening and evaluation of undernutrition in the elderly, especially in resource-limited environments. Given the scarcity of local data, our findings serve as important reference values for body composition and undernutrition among Senegalese elderly. We recommend that future research further investigates the calibration of diagnostic instruments—including BIA—against gold standards specifically in African populations, with the goal of optimizing both sample size determination and diagnostic accuracy in this context. The use of enhanced exploratory tools, such as box-plots described by Tukey (1977), would also aid in visualizing the distributional features of key variables, thus informing both research and clinical strategies.

## Ethical approval and participant consent

We have the opinion of the ethics committee of the CHU of FANN which authorizes all doctors in specialization to prepare their end of study dissertation

## Funding statement

Non applicable

## CRediT authorship contribution statement

**Sandra J Bitchoka Mbea:** Writing – original draft, Validation, Software, Methodology, Investigation, Data curation, Conceptualization. **Maïmouna Touré:** Writing – review & editing, Validation, Supervision, Resources, Conceptualization. **Claude Stephan Ohandza:** Writing – review & editing, Conceptualization. **Mamadou Coume:** Writing – review & editing, Project administration, Conceptualization.

## Declaration of competing interest

The authors declare that they have no known competing financial interests or personal relationships that could have appeared to influence the work reported in this paper.

## References

[1] (1998). National agency for accreditation and evaluation in health (ANAES). Acta Endosc.

[2] Power L., Mullally D., Gibney E.R., Clarke M., Visser M., Volkert D. (2018).

[3] Diagne S.L.M., Ka O., Fall F. (2017). Risques de chute et de dénutrition chez des personnes âgées vivant à domicile à Dakar (Sénégal). Med Afr Noire En Ligne.

[4] Cortés-Aguilar R., Malih N., Abbate M., Fresneda S., Yañez A., Bennasar-Veny M. (2024). Validity of nutrition screening tools for risk of malnutrition among hospitalized adult patients: a systematic review and meta-analysis. Clin Nutr.

[5] Cederholm T., Jensen G.L., Correia M.I.T.D., Gonzalez M.C., Fukushima R., Higashiguchi T. (2019). GLIM criteria for the diagnosis of malnutrition - A consensus report from the global clinical nutrition community. Clin Nutr Edinb Scotl.

[6] HAS. Diagnosis of undernutrition in people aged 70 years and older. 2021.

[7] Ponti F., Santoro A., Mercatelli D., Gasperini C., Conte M., Martucci M. (2020). Aging and imaging assessment of body composition: from fat to facts. Front Endocrinol.

[8] Vilaça K.H.C., Paula F.J.A., Ferriolli E., Lima N.K.C., Marchini J.S., Moriguti J.C. (2011). Body composition assessment of undernourished older subjects by dual-energy x-ray absorptiometry and bioelectric impedance analysis. J Nutr Health Aging.

[9] Buffa R., Floris G., Marini E. (2023). Body composition assessment of undernourished older subjects by dual-energy X-ray absorptiometry and bioelectric impedance analysis (1). J Nutr Health Aging.

[10] Courville A.B., Yang S.B., Andrus S., Hayat N., Kuemmerle A., Leahy E. (2020). Body adiposity measured by bioelectrical impedance is an alternative to dual-energy x-ray absorptiometry in black Africans: the Africans in America Study. Nutr Burbank Los Angel Cty Calif.

[11] Ba M., Sall A., Djajhete R., Ba D., Coume M. (2023). Unité de court séjour gériatrique du CHNU de Fann (Dakar, Sénégal) : bilan d’étape après plus d’une année de fonctionnement. NPG Neurol - Psychiatr - Gériatrie.

[12] Woldekidan M.A., Haile D., Shikur B., Gebreyesus S.H. (2021). Validity of Mini nutritional assessment tool among an elderly population in Yeka sub-city, Addis Ababa, Ethiopia. South Afr J Clin Nutr.

[13] Andre M.B., Dumavibhat N., Ngatu N.R., Eitoku M., Hirota R., Suganuma N. (2013). Mini nutritional assessment and functional capacity in community-dwelling elderly in rural Luozi, Democratic Republic of Congo. Geriatr Gerontol Int.

[14] Cascio B.L., Logomarsino J.V. (2018). Evaluating the effectiveness of five screening tools used to identify malnutrition risk in hospitalized elderly: a systematic review. Geriatr Nur (Lond).

[15] Sanca L., Byberg S., Có C.C., da Costa G, Indami M., Rama L. (2024). Body composition analysis using bioelectric impedance in Bissau: reproducibility and level of agreement between two available devices. Pan Afr Med J [Internet].

[16] Vaduva P., Thibault R. (2021). Évaluation de l’état nutritionnel chez l’adulte. EMC - Endocrinol-Nutr.

[17] Galmes-Panades A.M., Konieczna J., Varela-Mato V., Abete I., Babio N., Fiol M. (2021). Targeting body composition in an older population: do changes in movement behaviours matter? Longitudinal analyses in the PREDIMED-Plus trial. BMC Med.

[18] Calcaterra L., Abellan van Kan G., Steinmeyer Z., Angioni D., Proietti M., Sourdet S. (2024). Sarcopenia and poor nutritional status in older adults. Clin Nutr Edinb Scotl.

[19] Norazman C.W., Adznam S.N., Jamaluddin R. (2020). Malnutrition as key predictor of physical frailty among Malaysian older adults. Nutrients.

